# Coarse particles and hospital admissions due to respiratory diseases in children. An ecological time series study

**DOI:** 10.1590/1516-3180.2017.0362080218

**Published:** 2018-06-25

**Authors:** Ana Cristina Gobbo César, Luiz Fernando Nascimento

**Affiliations:** I PhD. Assistant Professor, Instituto Federal de Educação Ciência e Tecnologia de São Paulo (IFSP), Campus Bragança Paulista (SP), Brazil.; II MD, PhD. Researcher, Postgraduate Program on Environmental Sciences, Universidade de Taubaté (UNITAU), Taubaté (SP), Brazil.

**Keywords:** Air pollutants, Particulate matter, Child health, Respiration diseases, Coarse particles

## Abstract

**BACKGROUND:**

Exposure to particulate matter (PM) is associated with hospitalizations due to respiratory diseases among children.

**DESIGN AND SETTING:**

An ecological time series study was carried out to identify the role of coarse fractions of particulate matter (PM_10-2.5_) in hospitalizations among children up to 10 years of age, in Piracicaba (SP) in the year 2015.

**METHODS:**

A generalized additive model of Poisson regression was used to estimate the risk of hospitalization due to acute laryngitis and tracheitis, pneumonia, bronchitis, bronchiolitis and asthma. Lags of 0 to 7 days were considered, and the model was adjusted for the temperature and relative humidity of the air and controlled for short and long-term exposure. Proportional attributable ratios, population-attributable fractions and hospital costs were calculated with increasing concentrations of these pollutants.

**RESULTS:**

638 hospitalizations were evaluated during this period, with a mean of 1.75 cases per day (standard deviation, SD = 1.86). The daily averages were 22.45 µg/m^3^ (SD = 13.25) for the coarse fraction (PM_10-2.5_) and 13.32 µg/m^3^ (SD = 6.38) for the fine fraction. Significant risks of PM_10-2.5_ exposure were only observed at lag 0, with relative risk (RR) = 1.012, and at lag 6, with RR = 1.011. An increase of 5 µg/m^3^ in the coarse fraction concentration implied an increase in the relative risk of hospitalizations of up to 4.8%, with an excess of 72 hospitalizations and excess expenditure of US$ 17,000 per year.

**CONCLUSIONS:**

This study showed the impact of coarse-fraction exposure on hospital admissions among children due to respiratory diseases.

## INTRODUCTION

Air pollution is estimated to be directly responsible for more than two million deaths per year worldwide, caused by damage to the lungs and respiratory tract. A large proportion of the morbidity and mortality due to respiratory diseases is caused by exposure to particulate matter (PM).^1^^,^^2^

Particulate matter with an aerodynamic diameter of less than 10 µm (PM_10_) is composed of solid and liquid particles suspended in the air. It varies in size and chemical composition and is able to carry adsorbed chemicals, biological material and metals on its surface. The effects of exposure to fine particulate matter on health, particularly with regard to hospitalizations due to respiratory diseases, have been described in some studies.^3^^,^^4^^,^^5^^,^^6^^,^^7^^,^^8^^,^^9^

The particles in PM_10_ are further classified based on their capacity for lung penetration, as follows: fine (PM_2.5_) if their diameter is not more than 2.5 µm; and coarse (PM_10-2.5_) if their diameter is between 2.5 µm and 10 µm. For the latter fraction, which is commonly formed by mechanical grinding and resuspension of solid material, no specific standard for air quality analysis has been adopted, and the general PM_10_ standard is used.^2^

However, recently, the difference between coarse and fine particles has become more explicitly appreciated. Separate measurements of fine-particle (PM_2.5_) concentration and coarse-particle (PM_10-2.5_) concentration have been included in studies, rather than measurements of PM_2.5_ and PM_10_. This has shown that, in contrast to the high correlation between PM_10_ concentration and PM_2.5_ concentration, there is often much less correlation between PM_2.5_ concentration and PM_10-2.5_ concentration. It should be noted that coarse-particle concentration is usually obtained by subtracting a direct measurement of PM_2.5_ concentration from a direct measurement of PM_10_ concentration.^10^

In toxicological studies, some evidence that the coarse fraction may be more inflammatory than PM_2.5_ has been reported.^11^^,^^12^^,^^13^ According to Adar et al.,^2^ the health implications of exposure to the coarse fraction are still poorly understood, given that most research has focused on fine particles.

The objective of this study was to investigate the association between exposure to the coarse fraction of particulate matter and hospitalizations due to respiratory diseases among children up to 10 years of age living in Piracicaba (SP).

## METHODS

### Study design 

An ecological time series study on children from zero to 10 years of age living in Piracicaba was conducted from January 1, 2015, to December 31, 2015. Daily indicators of hospitalization due to respiratory diseases were gathered, including data on acute laryngitis and tracheitis, pneumonia, bronchitis, bronchiolitis and asthma, using the International Classification of Diseases (ICD) 10^th^ revision (J04, J12-J18, J20-J21 and J45). These data were obtained from the Department of Informatics of the Brazilian National Health System (Departamento de Informática do Sistema Único de Saúde, DATASUS).^14^

The study used data from the municipality of Piracicaba (SP). This municipality, located at the coordinates 22º 43’ S and 47º 39’ W, at an altitude of 547 meters, is considered to be of medium size and covers approximately 1,400 km^2^. Its estimated population in 2016 was around 400,000 inhabitants. Its vehicle fleet was estimated to be around 250,000 cars, trucks and motorcycles and 13,000 buses in 2015.^15^ Piracicaba is an important city in the sugar and alcohol industry, which means that pollutants in this region come not only from burning of fuel in vehicles but also from burning of straw from sugarcane production.

The mean temperature and relative humidity of the municipality were obtained from the website of the Environmental Sanitation Company of the State of São Paulo (Companhia Ambiental do Estado de São Paulo, CETESB).^16^

The 24-hour average concentrations of the pollutants PM_2.5_ and PM_10_ were used to calculate the coarse fraction. Thus, the coarse fraction (PM_10-2.5_) was obtained by subtracting the PM_2.5_ concentration from the PM_10_ concentration.

### Statistical analysis

Descriptive analyses on the daily average, minimum and maximum values, standard deviation of the study variables and the respective percentage values for pollutants, in accordance with the PM_10_ concentration, were presented in table form. Pearson’s correlation test was used to evaluate the possible correlations between hospitalizations and daily levels of pollutants (PM_2.5_ and coarse fraction).

To investigate these effects, the generalized Poisson regression additive model (GAM) was used. In this, each pollutant was analyzed separately (unipollutant model), with adjustments for mean temperature, relative humidity, short-term exposure and long-term seasonality of exposure.

Since the effects of exposure to air pollutants can lead to hospitalization on the same day as exposure (lag 0) or on days subsequent to exposure, the impacts on the respiratory tract on the day of hospitalization (lag 0) and on the subsequent seven days (lag 1 to lag 7) were investigated.

To calculate the relative risk (RR) of hospitalizations, calculated with a 95% confidence interval, increments of 5 µg/m^3^ were chosen for each pollutant, using the formula: RR = [exp (5 × β)], where β is the value of the coefficient provided by the adjusted regression model for each pollutant.

The concepts of proportional attributable risk (PAR) and population-attributable fraction (PAF) were used to estimate the excess hospitalizations due to the increase in the concentrations of the fine and coarse fractions of the particulate material.

PAR is expressed through the equation PAR = [1-1/RR], where RR is the relative risk; and PAF is expressed through the equation PAF = [PAR (%) * N], where N is the total population studied. The mean hospital admission costs relating to respiratory diseases were obtained from DATASUS.^14^

The Statistica 7 software (StatSoft, Inc., Tulsa, OK, USA) and a 5% significance level were used for these analyses.

This project was approved by the Research Ethics Committee of the University of Taubaté, under number 314/04.

## RESULTS

A total of 638 hospitalizations due to respiratory diseases among children aged less than 10 years were evaluated during this period. The mean daily frequency of hospitalization was 1.75 cases (standard deviation, SD = 1.86), with a range from zero to 10 cases per day. In relation to the pollutants, the daily averages were 13.32 µg/m^3^ (SD = 6.38) for the fine fraction and 22.45 µg/m3 (SD = 13.25) for the coarse fraction (PM_10-2.5_). These values were significantly different (P < 0.01). A high proportion of PM_10-2.5_ (62%) was found in the composition of PM_10_. These data are shown in Table 1.

**Table 1:** Descriptive analysis on the daily averages, minimum and maximum values and standard deviations of the study variables and the respective percentage values for pollutants. Piracicaba (SP), 2015

Variables	Mean (SD)	% ^(^*^)^	Minimum-maximum	%
PM_2.5_ (µg/m^3^)	13.3 (6.4)	38.1 (7.1)	3-41	20.7-57.9*
PM_10-2.5_ (µg/m^3^)	22.5 (13.3)	61.9 (7.1)	5-85	42.1-79.3
Mean temperature (ºC)	23.2 (3.2)	-	14.8-31.2	-
Relative humidity (%)	48.3 (14.6)	-	13-88	-
Hospital admissions	1.8 (1.9)	-	0-10	-

SD = standard deviation. *in accordance with PM_10_ concentration.

The period with the highest number of hospitalizations was from March to August. There were 59-113 hospitalizations per month during this period, representing 77% of all cases in the year 2015. This period coincides with the months during which sugarcane straw is burned and with the months when the weather is cooler.

Pearson correlation tests on the selected variables showed that the hospitalizations were positively and significantly correlated (P < 0.05) with the PM_2.5_ and PM_10-2.5_ concentrations. Regarding the mean temperature, hospitalizations showed significant negative correlations (P < 0.01), thus demonstrating that low temperatures may be associated with hospitalizations (Table 2).

**Table 2:** Pearson correlations for pollutants, mean temperature (MT), air relative humidity (RH) and hospitalizations (HA). Piracicaba (SP), 2015

	PM_2.5_	PM_10-2.5_	MT	RH	HA
PM_2.5_	1	0.84**	0.11*	-0.61**	0.13*
PM_10-2.5_		1	0.06	-0.64**	0.18*
MT			1	-0.28**	0.30**
RH				1	-0.01
HA					1

*P < 0.05; **P < 0.01.

The regression coefficients and the respective standard errors for the concentrations of PM_10-2.5_ and PM_2.5_ at each lag time, with adjustments for mean temperature, relative humidity, short-term exposure (days of the week) and long-duration seasonality of exposure (annual) are shown in Table 3.

**Table 3:** Poisson regression coefficients and standard errors (SE; in parentheses) for the fine and coarse fractions according to the lag time. Piracicaba (SP), 2015

Lag	Coarse fraction (SE)	Fine fraction (SE)
0	0.01158 (0.00402)^#^	0.01083 (0.00785)
1	0.00425 (0.00420)	0.00577 (0.00825)
2	0.00245 (0.00446)	-0.00169 (0.00864)
3	0.00586 (0.00449)	0.01185 (0.00854)
4	0.00402 (0.00423)	0.00986 (0.00860)
5	0.00136 (0.00415)	0.00429 (0.00846)
6	0.01107 (0.00401)^#^	0.01265 (0.00814)
7	0.00373 (0.00411)	-0.01270 (0.00841)

^#^P < 0.05. SE = standard error.

**Figure 1:**
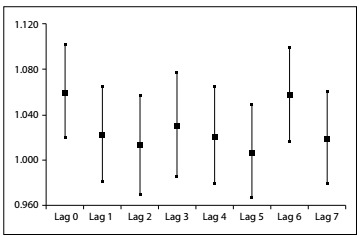
Increased relative risks (RR) and corresponding 95% confidence intervals for all time lags, after an increase of 5 µg/m^3^ in the concentration of PM_10-2.5_, Piracicaba, SP, 2015.

The significative relative risks of hospitalizations due to PM_10-2.5_ exposure were RR = 1.012 (95% confidence interval, CI: 1.004-1.020) at lag 0 and RR = 1.011 (95% CI: 1.003-1.019) at lag 6 (Figure 1). There was no significant association between exposure to the fine fraction and admissions, at any lag. Moreover, at lags 0 and 6, there was also no association between exposure to PM_10_ and admissions (data not shown). An increase of 5 µg/m^3^ in the coarse-fraction concentration implied increases in the relative risk of hospitalizations of 4.8% and 4.6% for lag 0 and lag 6, respectively.

A reduction of 5 µg/m³ in the coarse-fraction concentration would lead to a decrease of 73 hospitalizations, with a reduction in costs of around R$ 52,000 over the period analyzed, considering R$ 710.61 to be the mean cost of each hospitalization for the public healthcare system.

## DISCUSSION

The results from this study, which to the best of our knowledge was the first of its kind to be conducted in Brazil, showed that exposure to the coarse fraction of particulate matter was associated with hospitalizations due to respiratory diseases among children up to 10 years of age, both among hospitalizations on the same day as exposure (lag 0) and among hospitalizations six days after exposure (lag 6). The proportions between PM_2.5_ and PM_10_ found in the present study were of the order of approximately 38%, which differed from what has been reported in the literature (between 50 and 70%).^16^

There is evidence showing that short-term exposure to PM_10_ and long-term exposure to PM_2.5_ have effects involving increased mortality due to respiratory diseases. This demonstrates that the presence of both classes of particulate matter constitutes risk factors, even though greater emphasis has been placed on PM_2.5_ in the worldwide literature and presence of PM_2.5_ is considered to be more incisive in relation to emergence of respiratory diseases. It has been estimated that all-cause mortality (i.e. not only respiratory mortality) increases by 0.2-0.6% in response to an increase of 10 µg/m^3^ of PM_10_.^17^ In Brazil, the maximum concentrations of particulate matter (PM_10_) in six metropolitan areas of the country were above the limits established by the World Health Organization (WHO) in all years from 1995 to 2012, although these levels were within the limits established by Brazilian governmental agencies.^18^

Although the health implications of exposure to the coarse fraction (PM_10-2.5_) remain poorly explored and known, there seems to be an association between short-term exposure to this fraction and increased respiratory morbidity, especially as shown in daily averages in a meta-analysis performed by Adar et al.^2^

On the other hand, in a review article covering the period from 1995 to 2015, Froes Asmus et al.^18^ showed that 17 ecological time series studies carried out in medium-sized and large Brazilian municipalities have been published and that all of them correlated increased numbers of hospital admissions due to respiratory diseases among children with increased concentrations of atmospheric pollutants, but that in none of these articles was exposure to the coarse fraction of particulate matter considered. This shows the importance of the results presented in our study.

Cesar et al.,^3^ in Piracicaba (SP), used pollutant concentrations that were estimated through mathematical modeling (rather than from measurements) for the years 2011-2012. They observed that an increase in PM_2.5_ of 10 µg/m³ implied an increase in the relative risk of respiratory morbidity among children aged zero to 10 years, between August 1, 2011, and July 31, 2012, of between 7.9% (lag 1) and 8.6% (lag 3). The mean concentration of PM_2.5_ was estimated at 28.6 µg/m³ and the diseases studied were pneumonia and asthma.

In the present study, the data on the pollutants in the same city did not show any association between PM_2.5_ and hospitalizations due to respiratory diseases among children. Comparing our 2015 data with the 2011-2012 data from Cesar et al.,^3^ we observed that our mean PM_2.5_ value (13.3 µg/m³) was approximately 50% of the mean PM_2.5_ value in their study. We may speculate that the lack of association in our study may have been due to this lower PM_2.5_ value in 2015, compared with 2011-2012. However, when the possible association of the role of exposure to the coarse fraction in hospitalizations due to some respiratory diseases was studied, associations were found at lags 0 and 6 with relative risks of approximately RR = 1.012. Although these isolated associations at lags 0 and 6 may have been due to chance, the effects of exposure to air pollutants usually occur around the fourth or fifth day. Nonetheless, the possibility that this effect might occur on the same day as the exposure cannot be ruled out.

Research on the effects of burning biomass have shown that this leads to impairment of respiratory functions especially among younger children.^19^^,^^20^^,^^21^ In the city of Araraquara (SP), Souza and Nascimento^22^ showed that an increase of 10.0 µg/m^3^ in the concentration of PM_10_ led to increases in the relative risk of hospitalizations among children up to 10 years of age of 15.0% at lag 0 and 7.0% at lag 1. These values were higher than what was obtained in the present study through analysis on the coarse fraction. The previous study used data from an earlier period (2010 to 2012) before the implementation of state laws to control the burning of sugarcane straw, which suggests that these laws have had a positive impact over recent years.

Souza et al.^23^ conducted a study in Vitoria (ES) on daily hospital admissions of children under six years of age according to data on daily concentrations of air pollutants, including PM_10_, that were collected in automatic monitoring stations. They showed that an increase of 10 µg/m^3^ (interquartile range) in the levels of this pollutant resulted in a 3.0% increase in the relative risk of hospitalization.

Other differences between these two fractions relate to the longer half-life of the fine fraction (of the order of days to weeks), in contrast to the half-life of the coarse fraction (of the order of a few hours), and the greater distance that the fine fraction can reach, in comparison with the coarse fraction.^24^

Separate analysis on the fine and coarse fractions was justified in a study carried out in São José dos Campos (SP) where the compositions of these fractions were different. Ion concentrations differed, with higher levels of SO_4_^2-^, NH_4_^+^ and K^+^ in the fine fraction and higher levels of Cl^-^, NO_3_^-^, Na^+^ and Ca^2+^ in the coarse fraction.^25^

It seems that there is greater production of hydroxyl radicals, increased production of cytokines by macrophages and easier stimulation of pulmonary macrophages to produce inflammatory mediators when there is exposure to the coarse fraction, in comparison with the fine fraction. Thus, this constitutes a difference in the mechanism of action between these two fractions.^10^

One limitation of this study may come from its exclusion of hospitalizations that were not within the National Health System (Sistema Único de Saúde, SUS). However, the present study is likely to have been representative of the Brazilian population because the majority of the population makes use of this service. Another limitation may relate to respiratory morbidities such as pneumonia, asthma and bronchitis among children, which may have been treated on an outpatient basis, without resulting in patient hospitalization, and thus such patients will not have been included in this study. In addition, cases of underreporting of respiratory diseases may also have occurred through errors in making diagnoses or in selecting the disease coding within the International Classification of Diseases (ICD), thus contributing towards some imprecise accounting of cases of morbidity. Despite the above, the DATASUS portal is an official source of the Brazilian Ministry of Health and the date available are accepted for use in epidemiological studies. Another possible limitation of this study is the monitoring of air pollutants during the study, since the daily concentrations of the pollutants (PM_2.5_ and PM_10_) obtained from CETESB stations were considered to be homogeneous for the whole municipality, without evaluating the individual exposures to the pollutants analyzed here, and these were used to calculate the coarse fraction of the particulate material. In addition, the PM_10-2.5_ data were not obtained directly, but were obtained from two quantified values, i.e. PM_10_ and PM_2.5_, which may have contained errors.

## CONCLUSION

The results showed the effects of the coarse fraction of particulate matter on hospitalizations due to respiratory diseases among children, on the day of exposure and subsequently, up to six days afterwards. Another important finding from this study was that the concentration of the coarse fraction was high in relation to the fine particulate material, thus differing from what has been reported in the literature. In addition, the differences in these fractions that were observed regarding the sources where they originate and their compositions, half-lives, distances traveled and properties suggest that these fractions should be analyzed separately.
